# Aggressive prostate cancer with somatic loss of the homologous recombination repair gene FANCA: a case report

**DOI:** 10.1186/s13000-019-0916-z

**Published:** 2020-01-13

**Authors:** Hiroshi Hongo, Takeo Kosaka, Eriko Aimono, Hiroshi Nishihara, Mototsugu Oya

**Affiliations:** 1 Department of Urology, Keio University School of Medicine, 35 Shinanomachi, Shinjuku-ku, Tokyo, 160-8582 Japan; 2 Genomics Unit, Keio Cancer Center, Keio University School of Medicine, 35 Shinanomachi, Shinjuku-ku, Tokyo, 160-8582 Japan

**Keywords:** FANCA, Homologous recombination repair, Castration-resistant prostate cancer, Next-generation sequencing, Genomic analysis

## Abstract

**Background:**

Precision medicine based on genomic analysis of germline or tumor tissue is attracting attention. However, there is no consensus on how to apply the results of genomic analysis to treatment.

**Case presentation:**

A 59-year-old man diagnosed with metastatic prostate cancer was diagnosed with castration-resistant prostate cancer. Although he was sequentially treated with enzalutamide and abiraterone, bone metastasis progression was identified by skeletal scintigraphy. Therefore, we sequentially performed docetaxel therapy followed by cabazitaxel. After the third cycle of cabazitaxel, his prostate-specific antigen level was stable at < 10 ng/mL, and no radiological progression was detected.

The patient’s formalin-fixed paraffin-embedded tumor biopsy specimen underwent multiple-gene testing by next-generation sequencing, which identified a FANCA homodeletion. No significant germline mutation was observed.

**Conclusions:**

We describe a case of aggressive, castration-resistant prostate cancer with FANCA homodeletion. Genomic analysis of prostate cancer tissue can be useful to determine optimal treatment of such cancers.

## Introduction

Various drugs, such as second-generation antiandrogens, radium-223, and cabazitaxel, have been approved for treatment of castration-resistant prostate cancer (CRPC) in many countries, including Japan. However, the duration of response to these drugs is limited to several months. Although precision medicine based on genomic analysis of germline or tumor tissue is attracting attention, there is no consensus on how to apply the results of genomic analysis to treatment.

Allelic imbalance of 16q, which includes FANCA gene, is a known risk factor for cancer development or progression [1–3]. Recent studies have shown that DNA damage repair gene variants are biomarkers for the response to poly (ADP)-ribose polymerase (PARP) inhibitors [4] but are poor prognostic factors for prostate cancer [5].

We present a case of prostate cancer that was resistant to second-generation antiandrogens and taxanes and showed somatic loss of the homologous recombination repair gene FANCA.

## Case presentation

**Fig. 1:**
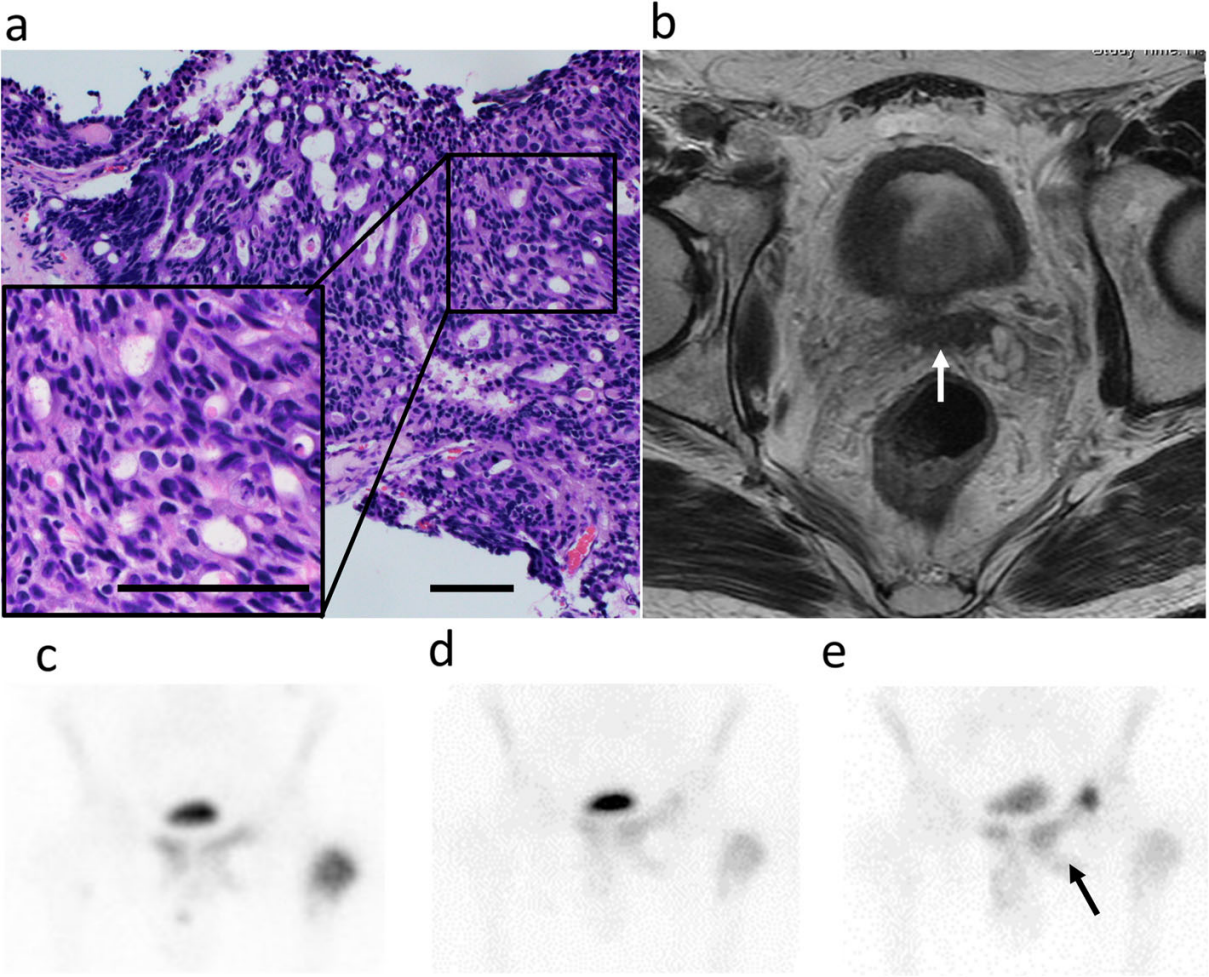
**a** Representative figure showing the hematoxylin and eosin staining of the prostate needle-biopsy specimens. The enlarged picture shows the tumor with Gleason patterns 4 and 5. Scale bar, 100 μm. **b** Magnetic resonance imaging (MRI) showing a prostatic tumor invading the seminal vesicles (arrow). **c** Skeletal scintigraphy at diagnosis. **d** Skeletal scintigraphy after the sixth cycle of docetaxel. **e** Skeletal scintigraphy 5 months after enzalutamide was started. Bone metastases were exacerbated (arrow)

**Fig. 2:**
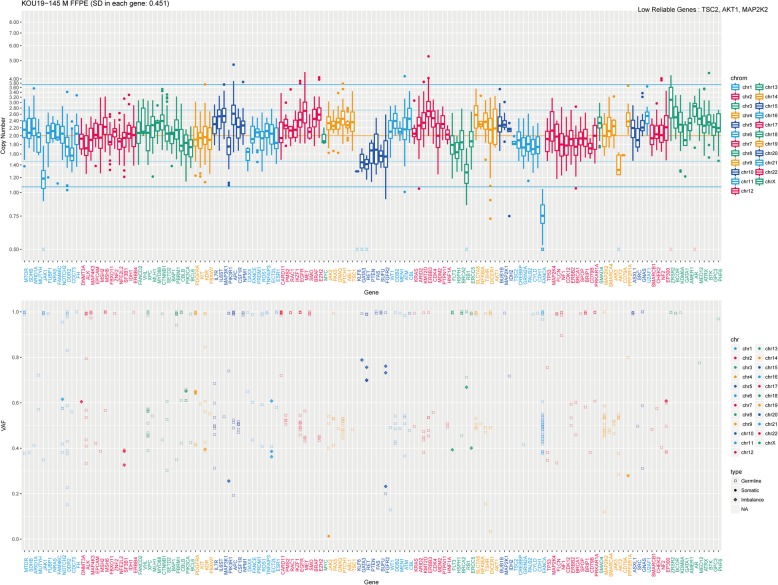
**a** The horizontal axis corresponds to the examined genes, and the vertical axis corresponds to the copy number. **b** The horizontal axis corresponds to the examined genes, and the vertical axis corresponds to the variant allele frequency

A 59-year-old man visited our hospital in July 2017 with an elevated level (88 ng/mL) of prostate-specific antigen (PSA). He was diagnosed with prostatic adenocarcinoma with a Gleason score of 4 + 5 = 9 (Fig. 1a) by prostate needle biopsy. Magnetic resonance imaging (MRI) showed a prostate tumor invading the seminal vesicles (Fig. 1b), and skeletal scintigraphy showed multiple bone metastases, including the pubis, ischium, and left femur (Fig. 1c). He started therapy with a gonadotropin-releasing hormone (GnRH) antagonist, and subsequently, docetaxel was added to the therapy for high-volume tumors. Although his PSA level reduced to 3.37 ng/mL, it started to increase gradually after the sixth cycle of docetaxel, and he exhibited gross hematuria at two months of treatment. MRI revealed progressive prostate cancer invading the bladder. He started enzalutamide, and his PSA level reduced from 7.08 to 3.16 ng/mL (55% reduction); however, progression of bone metastases was detected by skeletal scintigraphy after 5 months (Fig. 1d, e). Therefore, we sequentially started cabazitaxel therapy. His PSA level was stable, and no radiological progression was detected after the third cycle of cabazitaxel (Additional file 1). Acquiring cabazitaxel resistance was thought to be inevitable. Genomic analysis of the tumor and germline genome was performed because of patient’s concern about the heritability of the condition to his sons. We performed genomic analysis using both prostate needle-biopsy tissue for somatic aberration and white blood cells for germline aberration (Additional file 2). Next-generation sequencing identified homodeletion of FANCA in the tumor tissue. No significant germline mutation of FANCA was identified in white blood cell genome. Based on a copy number variations box plot and variant allele frequency plot (Fig. 2), the cancer had large subchromosomal deletions and allelic imbalance, which are reported to be found in homologous-recombination-impaired cancers [6].

## Discussion

DNA double-strand breaks are a serious threat to cell survival because they lead to a loss of chromosomal content. There are two main repair pathways for double-strand breaks: nonhomologous end joining and homologous recombination. FANCA belongs to the Fanconi anemia complementation group (FANC) family and is known as one of the genes responsible for Fanconi anemia [7]. It plays an important role in DNA interstrand crosslinking in homologous recombination repair [8].

Loss of FANCA function is associated with hereditary breast and ovarian cancer [9, 10]. FANCA variants are a significant risk factor for breast cancer among the population without BRCA1/2 loss [9]. Furthermore, loss of FANCA is associated with a familial history of prostate cancer [11, 12]. The National Comprehensive Cancer Network prostate cancer guideline recommends genetic counseling for patients with prostate cancer and having BRCA1/2, ATM, PALB2, or FANCA mutation [13].

Recently, the Gleason grade groups based on pathological findings has been considered as a prognostic factor for prostate cancer [14]. The World Health Organization has accepted this grading system since 2016 [15]. In our case, the Gleason grade group was 5, with an expected poor prognosis. A previous study reported that the prevalence of DNA repair mutation involving FANCA was higher in prostate cancer cases with high Gleason grade groups than in cases with low Gleason grade groups [16].

While germline loss of FANCA function is known as a causative variant for prostate cancer development, it has also been reported that somatic variants in DNA repair genes, including FANCA, are increased in metastatic CRPC tissue [17, 18]. Our patient had no pathogenic variants in the germline genome, and FANCA loss was considered an acquired variant. In light of these facts, we should investigate the genome not only of white blood cells but also of primary and recurrent tumors, because genes for homologous recombination repair pathways can be mutated independently of the germline.

Ovarian cancer cells with disruption of the FANC-BRCA2 pathway are highly sensitive to cisplati n[19]. In prostate cancer cells, FANCA knockout is associated with hypersensitivity to cisplatin [20]. In a phase 2 trial, CRPC cases with FANCA homodeletion tended to respond well to the PARP inhibitor olapari b[4]. Based on these findings, although we performed cabazitaxel therapy for bone metastasis progression, cisplatin-based chemotherapy or PARP inhibitors may be more effective for our patient.

## Conclusions

This report focuses on a case of aggressive CRPC with FANCA homodeletion. Cisplatin-based chemotherapy or PARP inhibitors can be an optimal treatment for CRPC with deficiency in the homologous recombination pathway.

## Supplementary information

**Additional file 1: Figure S1.** Time course of the patient’s PSA level and treatment.

**Additional file 2:** Materials and Methods.

## Data Availability

Not applicable.

## Abbreviations

DIN: DNA integrity number
FFPE: Formalin-fixed paraffin-embedded
GnRH: Gonadotropin-releasing hormone
MRI: Magnetic resonance imaging
PARP: Poly (ADP)-ribose polymerase
PSA: Prostate-specific antigen

## References

[1] Cher ML, Ito T, Weidner N, Carroll PR, Jensen RH (1995). Mapping of regions of physical deletion on chromosome 16q in prostate cancer cells by fluorescence in situ hybridization (FISH). J Urol.

[2] Cleton-Jansen AM, Callen DF, Seshadri R, Goldup S, McCallum B, Crawford J, Powell JA, Settasatian C, van Beerendonk H, Moerland EW (2001). Loss of heterozygosity mapping at chromosome arm 16q in 712 breast tumors reveals factors that influence delineation of candidate regions. Cancer Res.

[3] Grundy PE, Breslow NE, Li S, Perlman E, Beckwith JB, Ritchey ML, Shamberger RC, Haase GM, D'Angio GJ, Donaldson M (2005). Loss of heterozygosity for chromosomes 1p and 16q is an adverse prognostic factor in favorable-histology Wilms tumor: a report from the National Wilms Tumor Study Group. J Clin Oncol.

[4] Mateo J, Carreira S, Sandhu S, Miranda S, Mossop H, Perez-Lopez R, Nava Rodrigues D, Robinson D, Omlin A, Tunariu N (2015). DNA-repair defects and Olaparib in metastatic prostate Cancer. N Engl J Med.

[5] Mateo J, Cheng HH, Beltran H, Dolling D, Xu W, Pritchard CC, Mossop H, Rescigno P, Perez-Lopez R, Sailer V (2018). Clinical outcome of prostate Cancer patients with Germline DNA repair mutations: retrospective analysis from an international study. Eur Urol.

[6] Konstantinopoulos PA, Ceccaldi R, Shapiro GI, D'Andrea AD (2015). Homologous recombination deficiency: exploiting the fundamental vulnerability of ovarian Cancer. Cancer Discov.

[7] Kimble DC, Lach FP, Gregg SQ, Donovan FX, Flynn EK, Kamat A, Young A, Vemulapalli M, Thomas JW, Mullikin JC (2018). A comprehensive approach to identification of pathogenic FANCA variants in Fanconi anemia patients and their families. Hum Mutat.

[8] McMahon LW, Sangerman J, Goodman SR, Kumaresan K, Lambert MW (2001). Human alpha spectrin II and the FANCA, FANCC, and FANCG proteins bind to DNA containing psoralen interstrand cross-links. Biochemistry..

[9] Litim N, Labrie Y, Desjardins S, Ouellette G, Plourde K, Belleau P, Durocher F (2013). Polymorphic variations in the FANCA gene in high-risk non-BRCA1/2 breast cancer individuals from the French Canadian population. Mol Oncol.

[10] Ganzinelli M, Mariani P, Cattaneo D, Fossati R, Fruscio R, Corso S, Ricci F, Broggini M, Damia G (2011). Expression of DNA repair genes in ovarian cancer samples: biological and clinical considerations. Eur J Cancer.

[11] Pritchard CC, Mateo J, Walsh MF, De Sarkar N, Abida W, Beltran H, Garofalo A, Gulati R, Carreira S, Eeles R (2016). Inherited DNA-repair gene mutations in men with metastatic prostate Cancer. N Engl J Med.

[12] Hayano T, Matsui H, Nakaoka H, Ohtake N, Hosomichi K, Suzuki K, Inoue I (2016). Germline variants of prostate Cancer in Japanese families. PLoS One.

[13] NCCN Clinical Practice Guidelines in Oncology: Prostate Cancer. Version 4.2019 [ NCCN.org ] available at https://www.nccn.org/professionals/physician_gls/pdf/prostate.pdf.

[14] Pierorazio PM, Walsh PC, Partin AW, Epstein JI (2013). Prognostic Gleason grade grouping: data based on the modified Gleason scoring system. BJU Int.

[15] Epstein JI, Egevad L, Amin MB, Delahunt B, Srigley JR, Humphrey PA (2016). The 2014 International Society of Urological Pathology (ISUP) consensus conference on Gleason grading of prostatic carcinoma: definition of grading patterns and proposal for a new grading system. Am J Surg Pathol.

[16] Marshall CH, Fu W, Wang H, Baras AS, Lotan TL, Antonarakis ES (2019). Prevalence of DNA repair gene mutations in localized prostate cancer according to clinical and pathologic features: association of Gleason score and tumor stage. Prostate Cancer Prostatic Dis.

[17] Abida W, Armenia J, Gopalan A, Brennan R, Walsh M, Barron D, Danila D, Rathkopf D, Morris M, Slovin S, et al. Prospective genomic profiling of prostate Cancer across disease states reveals Germline and somatic alterations that may affect clinical decision making. JCO Precis Oncol. 2017;2017. 10.1200/PO.17.00029 PMC5558263 28825054

[18] Robinson D, Van Allen EM, Wu YM, Schultz N, Lonigro RJ, Mosquera JM, Montgomery B, Taplin ME, Pritchard CC, Attard G (2015). Integrative clinical genomics of advanced prostate Cancer. Cell.

[19] Taniguchi T, Tischkowitz M, Ameziane N, Hodgson SV, Mathew CG, Joenje H, Mok SC, D'Andrea AD (2003). Disruption of the Fanconi anemia-BRCA pathway in cisplatin-sensitive ovarian tumors. Nat Med.

[20] Beltran H, Eng K, Mosquera JM, Sigaras A, Romanel A, Rennert H, Kossai M, Pauli C, Faltas B, Fontugne J (2015). Whole-exome sequencing of metastatic Cancer and biomarkers of treatment response. JAMA Oncol.

