# Supplementary figures and images for: Aggressive prostate cancer with somatic loss of the homologous recombination repair gene FANCA: a case report
Source: Diagn Pathol. 2020 Jan 13;15:5. doi: 10.1186/s13000-019-0916-z (PMC6958728; doi:10.1186/s13000-019-0916-z)

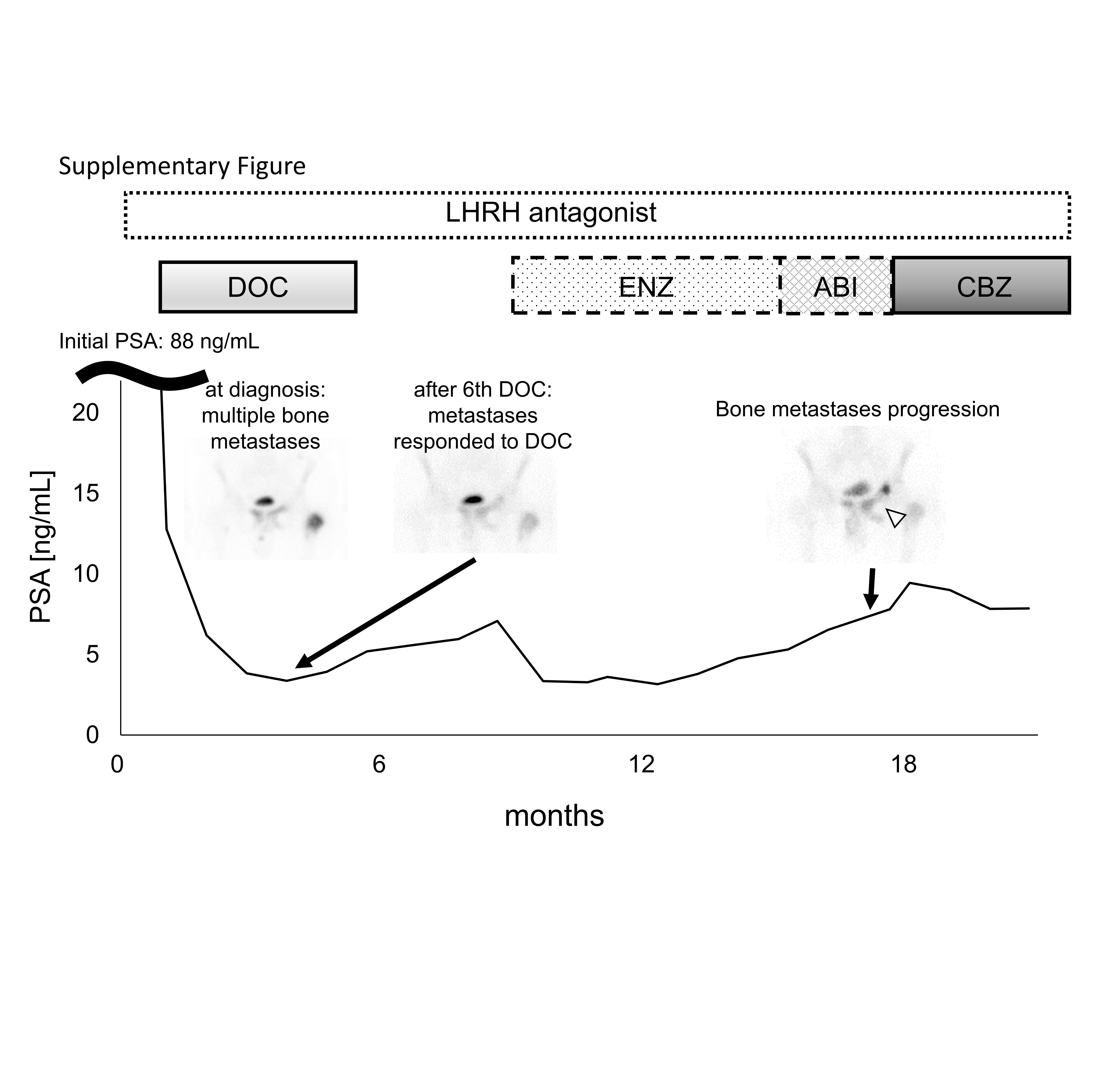

Supplement: Supplementary file 1 — Additional file 1: Figure S1. Time course of the patient’s PSA level and treatment. [file 13000_2019_916_MOESM1_ESM.tif]
