# Supplementary material for: Aggressive prostate cancer with somatic loss of the homologous recombination repair gene FANCA: a case report
Source: Diagn Pathol. 2020 Jan 13;15:5. doi: 10.1186/s13000-019-0916-z (PMC6958728; doi:10.1186/s13000-019-0916-z)
Supplement: Supplementary file 2 — Additional file 2: Materials and Methods. [file 13000_2019_916_MOESM2_ESM.docx]

**Materials and Methods**

Genomic DNA from both tumor tissue and peripheral blood mononuclear cells was extracted and purified. The minimum amount of DNA was 50 ng. We checked the quality of the DNA based on the DNA integrity number (DIN) score calculated using the Agilent 2000 TapeStation (Agilent Technologies, Waldbronn, Germany), and then we prepared DNA libraries for genome sequencing if the quality of DNA had a DIN score over 3.1. Subsequently, we performed targeted amplicon exome sequencing of 160 cancer-related genes using the Illumina MiSeq sequencing platform (Illumina, San Diego, CA, USA). Genome annotation and curation for analyzing the sequencing data were performed using an original bioinformatics pipeline called GenomeJack (Mitubishi Space Software, Tokyo, Japan) within three working days. We identified cancer-specific somatic gene alterations, such as single nucleotide variations, insertions/deletions, and copy number variations. The tumor mutation burden was calculated from these results. The analysis reports were discussed and reviewed in a genome expert conference of medical oncologists, molecular oncologists, pathologists, medical geneticists, clinical laboratory technicians, bioinformaticians, genetic counselors, pharmacists, and nurses. The final report, including information regarding the recommended treatment based on genomic profiling, was confirmed after approval at this conference. Subsequently, the report was returned to the physicians and patient.
